# Underlying construct of empathy, optimism, and burnout in medical students

**DOI:** 10.5116/ijme.54c3.60cd

**Published:** 2015-01-29

**Authors:** Mohammadreza Hojat, Michael Vergare, Gerald Isenberg, Mitchell Cohen, John Spandorfer

**Affiliations:** Sidney Kimmel (formerly Jefferson) Medical College at Thomas Jefferson University, Philadelphia, USA

**Keywords:** empathy, optimism, burnout, medical students

## Abstract

**Objectives:**

This study was designed to explore the underlying construct of measures of empathy, optimism, and burnout in medical students.

**Methods:**

Three instruments for measuring empathy (Jefferson Scale of Empathy, JSE); Optimism (the Life Orientation Test-Revised, LOT-R); and burnout (the Maslach Burnout Inventory, MBI, which includes three scales of Emotional Exhaustion, Depersonalization, and Personal Accomplishment) were administered to 265 third-year students at Sidney Kimmel (formerly Jefferson) Medical College at Thomas Jefferson University. Data were subjected to factor analysis to examine relationships among measures of empathy, optimism, and burnout in a multivariate statistical model.

**Results:**

Factor analysis (principal component with oblique rotation) resulted in two underlying constructs, each with an eigenvalue greater than one. The first factor involved “positive personality attributes” (factor coefficients greater than .58 for measures of empathy, optimism, and personal accomplishment). The second factor involved “negative personality attributes” (factor coefficients greater than .78 for measures of emotional exhaustion, and depersonalization).

**Conclusions:**

Results confirmed that an  association exists between empathy in the context of patient care and personality characteristics that are conducive to relationship building, and considered to be “positive personality attributes,” as opposed to personality characteristics that are considered as “negative personality attributes” that are detrimental to interpersonal relationships. Implications for the professional development of physicians-in-training and in-practice are discussed.

## Introduction

Empathy is defined as a cognitive attribute that involves understanding a patient’s suffering and concerns combined with an ability to communicate this understanding and an intention to help.^1^^-^^3^ It has also been described as the royal road to an optimal physician-patient relationship, and an essential component of overall physician competence.^1^^,^^2^  Empirical findings suggest that high scores on a validated instrument to measure empathy in the context of medical education and patient care (Jefferson Scale of Empathy, JSE)^3^ in medical students were associated with following positive outcomes: higher ratings of overall clinical competence given by medical school faculty,^4^ better interpersonal skills assessed by standardized patients,^5^ nomination by peers as positive influencers in the class,^6^^,^^7^ possessing professionalism attributes,^8^ greater interest in people-oriented (as opposed to technology- or procedure-oriented) specialties,^9^^,^^10^ leadership potential, ^6^ emotional intelligence, optimism and mood regulation,^11^ teamwork and interprofessional collaboration, positive attitudes toward integrative patient care,^12^ warmth, trust, tolerance, and dutifulness,^13^ and personality attributes that are conducive to relationship building.^7^

Optimism and burnout are also personal attributes that can influence medical students’ behavior and academic achievement. Optimism is defined as a powerful cognitive filter that can influence an individuals’ views of events and their adaptations and reactions to events.^14^ Academic and occupational burnout involves a psychological syndrome that includes emotional exhaustion and depersonalization that negatively influences academic and professional accomplishments.^15^

Despite the importance of empathy, optimism, and burnout, there is limited empirical research on the complex relationships among them in the context of medical education and patient care. In particular, with a few exceptions, empirical research on the association between empathy and optimism is difficult to find. For example, in a study of pediatric physicians and nurses, optimism was found as a significant predictor of the sense of a meaningful life.^16^ In another study of cognitive therapists and their patients, patient perceptions of therapists’ empathy predicted patients’ optimism.^17^

Burnout has been recognized as a significant contributing factor in suboptimal patient care.^18^^,^^19^ However, there is a dearth of empirical research on the association between empathy and burnout. For example, in a study with medical students^20^ an inverse relationship was observed between perceived burnout (measured by the Maslach Burnout Inventory, MBI^15^^,^^21^) and empathy (measured by the Interpersonal Reactivity Index, IRI^22^). Rosen and colleagues^23^ reported a decline in empathy (measured by IRI) but an increase in burnout (measured by MBI) from the beginning to the end of the internship year in a residency training program. Brazeau and colleagues^24^ reported that empathy scores (measured by the Jefferson Scale of Empathy, JSE^3^^,^^9^) in medical students were positively correlated with scores on personal accomplishment, but inversely correlated with scores on the emotional exhaustion and depersonalization scales of the MBI. The relationship between empathy and burnout has also been studied at the neurological level. In a functional magnetic resonance imaging (fMRI) study it was found that higher burnout scores (measured by the MBI) were predictive of reduced empathy-related brain activities.^25^ Beckman and colleagues^26^ reported that internal medicine residents’ empathy scores (measured by the JSE) were associated with residents’ positive views of faculty; but  no significant association was observed between burnout (measured by the MBI) and assessments of faculty.

Exploring the relationships among empathy, optimism, and burnout are important considering the findings that empathy tends to erode in undergraduate^27^ and graduate medical education;^28^ that burnout is highly prevalent among medical students (as high as 50%),^29^ residents (70%),^30^ and in physicians (30-40%),^31^ and that optimism is declining as a result of changes in the health care system in the United States.^32^

To our knowledge, in the previous studies the relationships among empathy, optimism, and burnout have not been explored in a multivariate statistical model by using a validated measure of empathy in the context of medical education and patient care. We designed this study to explore the underlying construct among empathy, optimism, and burnout scales in a multivariate statistical model.

## Methods

### Participants

Research participants included 265 third-year students at Sidney Kimmel (formerly Jefferson) Medical College at Thomas Jefferson University (49% women, *n*=130).

### Instruments

The following three instruments were used:

The Jefferson Scale of Empathy (JSE) was used for measuring empathy in the context of patient care (20 Likert items answered on a 7-point scale). We used the S-Version of the JSE in this study which was developed for administration to medical students. Evidence in support of the JSP’s validity and reliability has been reported.^3^^,^^4^^,^^9^^,^^10^^,^^13^ The JSE has been widely used by researchers in the United States and abroad, has been translated into 47 languages, and used in more than 70 countries.^33^ The possible score range is 20-140.

The Revised Life Orientation Test (LOT-R)^34^^-^^36^ was used for measuring generalized optimism. This instrument includes six Likert items (and four fillers), answered on a 5-point scale.  The possible score range is 5-30.

The Maslach Burnout Inventory (MBI)^15^^,^^21^^,^^37^^,^^38^ (Human Services Survey version, distributed by Mind Garden^©^) was used which includes 22 Likert items answered on a 6-point scale. The MBI includes three scales: Emotional Exhaustion (EE, 9 items intended to measure emotional exhaustion, possible score range 0-54), Depersonalization (DP, 5 items intended to measure perceptions of impersonal, non-appreciative responses by others for providing services or help, possible score range 0-30), and Personal Accomplishment (PA, 8 items, measuring perceptions of competence and successful academic and professional achievement, possible score range 0-48).

In reviewing the MBI, we noticed that some items needed minor modifications in wording to improve their face validity for administration to medical students. For example, “patients” was replaced for “recipients” in 7 items, such as the following: “I can easily understand how my recipients feel about things.”  Permission for these adaptations was obtained from the author of the inventory (e-mail response from Dr. Christina Maslach to MH, dated March 27, 2014).

### Procedures

This study which was approved by the University’s Institutional Review Board was conducted in March 2014. A packet of three instruments (JSE, LOT-R, and MBI) with a cover letter describing the purpose of the study as examining relationships between empathy, optimism, and burnout was distributed to the students during a class session on medical professionalism in the spring of the third medical school year. Students were assured about the confidentiality of individual data. Verified data from hard copies were transferred to an Excel file for statistical analysis. The Statistical Analysis System (SAS, 9.3 Version for Windows) was used for data analyses. Pearson correlations were calculated to examine bivariate relationships, and the principal component factor extraction method with oblique rotation was used to determine the underlying components of the five variables (Empathy, Optimism, Emotional Exhaustion, Depersonalization, and Personal Accomplishment) in a multivariate statistical model.

## Results

Of the total 282 students in the class, 265 returned completed surveys, representing a 94% response rate. Means and standard deviation for the five variables are reported in Table 1.

Exhaustion, Depersonalization, and Accomplishment were measured by the Maslach Burnout Inventory (MBI^15^^, ^^21^^,^^37^^,^^39^). Mean score and standard deviation of the JSE were in the range found in most other studies with medical students. ^4^^,^^5^^,^^7^^,^^8^^,^^12^ Mean scores of the scales of the emotional exhaustion, depersonalization, and personal accomplishment of the MBI scales were all in range of moderate scores for those scales.^39^ (Scores between 17-26 on emotional exhaustion, scores between 7-12 on depersonalization, and scores between 32-38 on personal accomplishment are considered moderate range).^39^ Examination of bivariate correlations showed that empathy scores were significantly and positively correlated with scores of personal accomplishment of the MBI (r=.36, p < .01), but inversely correlated with scores of depersonalization of the MBI (r=-.25, p <.01). Optimism scores were significantly and positively correlated with scores of personal accomplishment of the MBI (r=.36, p <.01), but negatively correlated with scores of emotional exhaustion scale of the MBI (r=-.35, p <.01). Also, scores on the emotional exhaustion scale were correlated negatively with those of personal accomplishment of the MBI (r=-.24, p <.01). Finally, scores of emotional exhaustion and depersonalization were positively correlated (r=.46, p <.01).

Summary results of factor analysis are reported in Table 2. As shown in the table, two factors emerged, each with an eigenvalue greater than one (1.9 and 1.1, respectively). The two factors accounted for 61% of the total variance (39% for the first factor; 22% for the second factor). Desirable personality attributes such as empathy, optimism, and personal accomplishment had substantial factor coefficients (≥ .58) under the first factor, which was entitled as a construct involving “positive personality attributes.”  Conversely, undesirable personality attributes such as emotional exhaustion and depersonalization had substantial factor coefficients (≥ .78) under the second factor, which was entitled as a construct involving “negative personality attributes.” Correlation between the two factors was -.22.

**Table 1:** Means, Standard Deviations of Empathy, Optimism, and the Maslach Burnout Inventory Scales (exhaustion, depersonalization, and accomplishment)

Variables*	M (SD)
Empathy	111.9 (11.2)
Optimism	22.2 (4.9)
Exhaustion	25.1 (9.9)
Depersonalization	9.1 (5.3)
Accomplishment	33.4 (6.1)

*Empathy was measured by the Jefferson Scale of Empathy (JSE, S-Version, refs: 3,4,9,10,13). Optimism was measured by the Revised Life Orientation Test (LOT-R34-36). Exhaustion, Depersonalization, and Accomplishment were measured by the Maslach Burnout Inventory (MBI, refs: 15, 21,37,39).

**Table 2:** Summary Results of factor Analysis* of scores of the Jefferson Scale of Empathy, Optimism, and the Maslach Burnout Inventory Subscales (exhaustion, depersonalization, and accomplishment)

Variables	Factor 1		Factor 2
Empathy	**.58**		- .07
Optimism	**.64**		-.13
Exhaustion	-.18		**.78**
Depersonalization	.10		**.90**
Accomplishment	**.89**		-.12
Eigenvalue	1.9		1.1
% Variance	39%		22%

*Principal component factor extraction with oblique rotation. Factor coefficients higher than .55 are shown in bold. Factor 1 was entitled as a construct involving “positive personality attributes” and Factor 2 as a construct involving “negative personality attributes.”

## Discussion

Findings of this study suggest that in a bivariate statistical analysis, empathy is positively correlated with personal accomplishment, but inversely associated with an indicator of burnout (depersonalization scale of the MBI). Higher optimism scores were associated with higher scores on personal accomplishment and with lower scores on emotional exhaustion of the MBI. Findings regarding positive correlation between scores on the emotional exhaustion and depersonalization scales and negative correlation between scores on the emotional exhaustion and personal accomplishment scales of the MBI are consistent with those reported in a meta-analytic study of the MBI.^38^ 

The bivariate statistical analysis is informative, but it presents a simplistic view of a complex relationship among multiple variables. A more comprehensive picture of the relationships was depicted when all variables were simultaneously taken into consideration in a multivariate statistical model by using factor analysis. In such a multivariate statistical model that we used in this study, two constructs of positive and negative personality attributes emerged, suggesting that empathy, optimism, and personal accomplishment are elements of a positive personality construct. Empirical research in which a direct link between validated measures of empathy and optimism has been examined is difficult to find. However, the relationships between empathy and optimism have been discussed at the conceptual level.^40^   Some studies provide indirect empirical evidence on variables that share commonality with both empathy and optimism. For example, Cohen and Hoffner^41^ found that empathic concern (measured by the IRI)^22^ predicted donation willingness. Meanwhile, Rodrigue and colleagues^42^ reported that registered organ donors expressed more optimism than non-registered persons. These findings suggest that willingness to donate has a commonality (or “share variance” in statistical terms) with both empathy and optimism. Thus, a link between measures of empathy and optimism could be expected, which was confirmed in our multivariate statistical analysis. 

Our findings are consistent with those reported by Zenasni and colleagues^43^ in their study of general practitioners in France, who observed significant but negative correlations between empathy factor scores (measured by the JSE), and scores on the depersonalization scale of the MBI. They also reported significantly positive correlations between empathy and personal accomplishment scores.^43^ Brazeau and colleagues^24^ reported that higher scores on the depersonalization and emotional exhaustion scales (measured by the MBI) were significantly associated with lower empathy scores (measured by the JSE) among medical students. Our findings in both bivariate and multivariate analyses confirmed the inverse relationships between scores on the empathy and depersonalization scales, but not between empathy and emotional exhaustion. Also, recent brain imaging findings that burnout in medical professionals could reduce empathy-related brain activities^25^ provides tangible evidence of the link between empathy and burnout in the human brain.

Findings of this study that empathy, optimism, and personal accomplishment are all interrelated elements of a positive personality suggest that if there are ways that any of these elements could be improved or safeguarded, a corresponding enhancement would be likely in the other elements, even without knowing the precise cause and effect relationships.  Thus, it could be expected that an improvement in empathy as a result of targeted empathy enhancement programs^44^^,^^45^ would increase medical students’ feelings of optimism and personal accomplishment. Krasner and colleagues^46^ reported that targeted educational programs in mindfulness, communication and self-awareness in primary care physicians could not only increase empathy (measured by the JSE) and personal accomplishment (measured by MBI) scores, but also contributed to decreasing scores on the emotional exhaustion and depersonalization scales of the MBI.

Nielsen and Tulinius^47^ presented evidence that burnout and compassion fatigue could be prevented in general practitioners by a simple intervention such as guided group discussion of stressful cases. Solar and colleagues^48^ observed that burnout was a common problem in family physicians in Europe and was detrimental to the well-being of physicians because of its association with job dissatisfaction and substance abuse. West and colleagues^49^ reported that higher levels of stress and burnout were associated with higher incidents of medical errors. These findings support the notion that enhancing empathy and optimism, and preventing burnout could have beneficial effects on medical students, physicians and patients.

We should exercise caution in the generalization of the findings of this study due to the single institution aspect of the study. This limitation, however, may be somewhat mitigated by the fact that Sidney Kimmel Medical College at Thomas Jefferson University is similar to other large private medical schools in the United States with regard to its four-year medical education curriculum, composition of student body, attrition rate, and career choices.

The strengths of the study include a relatively high response rate (94%), use of a validated measure of empathy which was specifically developed to measure empathy in the context of medical education and patient care, and using a multivariate statistical model for a more comprehensive analysis of complex relationships among measures of empathy, optimism, and burnout in medical students.

In summary, our findings that empathy, optimism, and burnout are intertwined in a larger picture will have implications for enhancing well-being of physicians-in-training and in-practice. Considering the potential for interventions in professional development of health care workers, our multivariate findings support previous findings that empathy in the context of patient care is associated with positive personality attributes that are conducive to relationship building, as opposed to negative personality attributes that are detrimental to interpersonal relationships.^7^^,^^8^^,^^12^ The multivariable findings may also suggest that an improvement in any of the studied variables (empathy, optimism, and burnout) could have a beneficial effect on any other variable. This leads to an important implication for professional development of health profession students and practitioners.

## 

### Acknowledgements

 The authors would like to thank Dorissa Bolinski for her editorial assistance.

### Conflict of Interest

The authors declare that they have no conflict of interest.
